# RETRACTION: Impact of Spinal Anaesthesia Versus General Anaesthesia on the Incidence of Surgical Site Infections After Knee or Hip Arthroplasty: A Meta‐Analysis

**DOI:** 10.1111/iwj.70672

**Published:** 2025-04-23

## Abstract

**RETRACTION:** LiZ. , XuX. , ZhuangZ. , LuJ. , GaoF. , and JiangQ. , “Impact of Spinal Anaesthesia Versus General Anaesthesia on the Incidence of Surgical Site Infections After Knee or Hip Arthroplasty: A Meta‐Analysis,” International Wound Journal 21, no. 1 (2024): e14369, 10.1111/iwj.14369.37649253 PMC10781890

The above article, published online on 30 August 2023, in Wiley Online Library (http://onlinelibrary.wiley.com/), has been retracted by agreement between the journal Editor in Chief, Professor Keith Harding; and John Wiley & Sons Ltd. Following an investigation by the publisher, all parties have concluded that this article was accepted solely on the basis of a compromised peer review process. The editors have therefore decided to retract the article. The authors disagree with the retraction.

**RETRACTION:** Z. Li , X. Xu , Z. Zhuang , J. Lu , F. Gao , and Q. Jiang , “Impact of Spinal Anaesthesia Versus General Anaesthesia on the Incidence of Surgical Site Infections After Knee or Hip Arthroplasty: A Meta‐Analysis,” International Wound Journal 21, no. 1 (2024): e14369, 10.1111/iwj.14369.37649253 PMC10781890

The above article, published online on 30 August 2023, in Wiley Online Library (http://onlinelibrary.wiley.com/), has been retracted by agreement between the journal Editor in Chief, Professor Keith Harding; and John Wiley & Sons Ltd. Following an investigation by the publisher, all parties have concluded that this article was accepted solely on the basis of a compromised peer review process. The editors have therefore decided to retract the article. The authors disagree with the retraction.

